# An Exploratory Study on the Complexity and Machine Learning Predictability of Stock Market Data

**DOI:** 10.3390/e24030332

**Published:** 2022-02-25

**Authors:** Sebastian Raubitzek, Thomas Neubauer

**Affiliations:** Information and Software Engineering Group, Institute of Information Systems Engineering, Faculty of Informatics, TU Wien, Favoritenstrasse 9-11/194, 1040 Vienna, Austria; thomas.neubauer@tuwien.ac.at

**Keywords:** hurst exponent, stock market data, time series prediction, machine learning, time series analysis, R/S analysis, Fisher’s information, Shannon’s entropy, fractal dimension, regression analysis, predictability, complexity

## Abstract

This paper shows if and how the predictability and complexity of stock market data changed over the last half-century and what influence the M1 money supply has. We use three different machine learning algorithms, i.e., a stochastic gradient descent linear regression, a lasso regression, and an XGBoost tree regression, to test the predictability of two stock market indices, the Dow Jones Industrial Average and the NASDAQ (National Association of Securities Dealers Automated Quotations) Composite. In addition, all data under study are discussed in the context of a variety of measures of signal complexity. The results of this complexity analysis are then linked with the machine learning results to discover trends and correlations between predictability and complexity. Our results show a decrease in predictability and an increase in complexity for more recent years. We find a correlation between approximate entropy, sample entropy, and the predictability of the employed machine learning algorithms on the data under study. This link between the predictability of machine learning algorithms and the mentioned entropy measures has not been shown before. It should be considered when analyzing and predicting complex time series data, e.g., stock market data, to e.g., identify regions of increased predictability.

## 1. Introduction

The topic of the efficient market hypothesis [1], i.e., if stock markets are predictable or not, is still a relevant topic today. Though there seems to be the agreement that stock market data are hard to predict, the efficient market hypothesis is still debated today, and one can find arguments for and against it.

In this research, we focus on the random walk aspect of the efficient market hypothesis, which is referred to as the *weak form* of the efficient market hypothesis [2]. The random walk theory says that the future evolution of prices cannot be predicted, i.e., that prices do not have memory. One of the most known authors of the 20th century on the efficient market hypothesis is Eugene Fama, who found evidence for the random walk character of stock markets based on empirical studies [3]. In the 1990s, though, many researchers contradicted the random walk hypothesis by emphasizing how investors behave, and the corresponding predictability of stock markets, as in [4]. Just as the name, *a non-random walk down wall street* suggests, the hypothesis is contradictory to its famous predecessor *a random walk down wall street* [5], an investment guide.

In the past, there were many attempts to show the predictability and efficiency of stock markets using the data’s inherent long-term memory or complexity, as in [6], where the Hurst exponent [7], was employed for this task. Here we hypothesize that a stock market’s complexity, which refers to any measure for non-linearity, signal-complexity, or noisiness, is crucial for its predictability and efficiency and must therefore be considered.

Furthermore, as discussed in [8], the available money supply, e.g., M1, influences stock market prices, and vice versa. The influence of the money supply on stock market data or cryptocurrencies and the corresponding inflation is evident (cf. https://inflationchart.com or https://fred.stlouisfed.org accessed on 17 January 2022). Thus we will also discuss the influence of money supply on the stock market data under study.

For our research, we revisit this topic of stock market data as a random walk. Therefore, we want to determine if there are trends in predictability and complexity for the stock market data understudy, if they correlate, and what influence inflation, i.e., an adjustment for the available money supply, has on the stock market data and its predictability further, if there is evidence that the stock market data under study is closer to a random walk, i.e., a fractional Brownian motion, for later years than for earlier years. Therefore, we use statistics, artificial intelligence, and complexity analysis tools to show if and how the predictability and complexity of stock market data changed over the last half-century and how the M1 money supply influences the predictability and complexity.

In Section 2, we discuss similar ideas and approaches from the past. An in-depth description of our approach, the data sets, and the employed techniques is given in Section 3. We show and discuss our findings in Section 4. We conclude our study in Section 5. We further collected some of our results in Appendix A and Appendix B to keep the main text focused.

## 2. Related Work

Our approach combines machine learning algorithms and measures of signal complexity/information. Therefore, we evaluate past approaches where these disciplines merged to analyze financial markets or related data. In most cases, the complexity of the studied time series is used to improve machine learning approaches or gain deeper insights into the dynamics of the time series data.

In [9], a new technique for calculating the fractal dimension of a time series is presented. Furthermore, this technique is combined with neural networks and fuzzy logic to make predictions for, e.g., the dollar/peso exchange rate.

The work of [10] analyzes the Nikkei stock prices for 1500 days. Fractal analysis is performed, and the corresponding Hurst exponent and fractal dimension are calculated. The fractal dimension and the Hurst exponent indicate a persistent behavior and, thus, the time series can theoretically be forecast. In addition, the strongest correlation was found for a period of three days, and so the input nodes of the machine learning approach were set to three days and compared to, e.g., five days, whereas the approach with three days outperformed the other ones.

In [11], the authors state that time series with a larger *Hurst exponent* can achieve higher accuracy when predicted using back-propagation neural networks than time series with a Hurst exponent close to 0.5. Thus, the Hurst exponent is calculated for 1024 trading day periods of the Dow-Jones index from 2 January 1930 to 14 May 2004. Afterward, these intervals are forecast, and results show that a time series with a higher Hurst exponent can be forecast more accurately than those with a lower Hurst exponent.

The work of [12] analyzes and predicts stock market closing prices using an enhanced evolutionary artificial neural network model. Further, R/S analysis is used to calculate the Hurst exponent for different scales and each time series data under study. This is used for identifying the regime of maximal persistency, i.e., where the Hurst exponent is maximal. These regimes were then used to tailor the input windows of the employed neural network model. The Hurst-based models did not outperform the regular ones; however, when employing the Hurst-improved model for trading strategies, the Hurst-improved ones outperformed the regular ones.

In [13], using the Hurst exponent, one can identify random walk patterns in a time series, i.e., with a Hurst exponent H≈0.5. Thus regions with H≉0.5 were identified and forecast using artificial neural networks, decision trees, and k-nearest neighbor models. Thus reaching an accuracy of up to 65%.

In [14], three different time series data are predicted using a NARX (nonlinear autoregressive model process with exogenous input) dynamic recurrent neural network. Two are chaotic time-series data, and the third is the BET (average of daily closed prices for nine representatives, most liquid listed companies at the Bucharest Stock Market) times series. Fractal analysis using the Hurst exponent is applied and indicates that all three are non-random, i.e., have a Hurst exponent of H≠0.5. The predictions are very good for the two chaotic time series, however the BET time series, despite a high Hurst exponent, is well below the others, as it is the only real-life time series data among the three.

In addition, in [15], the authors perform fractal analysis to exclude random behavior and to indicate predictability of the data under study. The stock indices understudy shows a persistent behavior, i.e., a Hurst exponent H>0.5. Afterward, machine learning methods (adaptive neuro-fuzzy inference system, dynamic evolving neuro-fuzzy inference system, Jordan neural network, support vector regression, and random forest) are used to predict future market development. The results show that these time series can, to some degree, effectively be forecast.

In [16], the authors intended to show the existence of a relationship between long term memory in time series data and the predictability of neural network forecasts of financial time series data. Brazilian financial assets traded at BM&FBovespa, specifically public companies shares and real estate investment funds, were analyzed using R/S analysis and the corresponding *Hurst exponent*. The study shows that one can achieve higher returns when considering time series with a higher Hurst exponent and neglecting an anti-persistent time series with a Hurst exponent H<0.5.

In [17], eight different stock market indices are analyzed using the *Hurst exponent*, Shannon entropy, and Rényi entropy. Additionally, time-dependent complexity features using these three complexity measures were added to each data set. Further, linear interpolation was used to augment the study data and generate larger data sets. Those data sets were then predicted using Multi-Layer Regression (MLR), Support Vector Regression (SVR), and feed forward back propagation models. The best results were obtained when using feed forward back propagation, including all three complexity features, i.e., Hurst exponent, Rényi entropy, and Shannon entropy.

Given the mentioned approaches, we want to use a wider variety of complexity measures to analyze financial time series data: In [18], approximate entropy, fractal dimension, and long term memory were used to test for market efficiency and [19] also uses approximate entropy to check for irregularities in financial data.

In [20], the authors give an overview of combined approaches of machine learning and measures of signal complexity for time series analysis, many references and methods discussed in the current article are presented in a wider context with an emphasis on how to combine these two areas of research.

Lastly, [21,22] provide evidence for the applicability of XGBoost to stock market data. Whereas, using a Lasso regression for stock market analysis, [23] employs linear regression to analyze stock market data.

There are various methods to choose from when it comes to predicting stock market exchange rates; for our purposes, we chose a LASSO regression, an XGBoost tree-based algorithm, and a common stochastic gradient descent linear regression method.

In [24], a LASSO regression is used to predict stock market data and for the featured application, outperforms other methods such as ridge linear regression or a Bayesian regularized artificial model. Furthermore, in [25,26], Lasso regression is employed for stock market analysis and prediction.

In [27], a variety of tree-based classifiers is used to predict stock market prices. The employed algorithms are random forest decision tree models and gradient boosted decision trees, such as XGBoost. In addition, in [28], XGBoost is used to forecast oil prices. Further, [22] uses an XGBoost algorithm to predict the direction of stock market data.

The work of [29] analyzes stock market data using several different algorithms, featuring a basic stochastic gradient descent linear regression model. Furthermore, in [23], a linear regression approach is used to predict stock market data.

## 3. Methodology

We developed the following procedure to test stock market data for its predictability:

1. Split data into sub-intervals; in our case, we split the data into annual sub-datasets, i.e., we treated each year separately.
2. We measured the signal complexity of each data set, i.e., each year, using the following complexity measures: Fisher’s information, Shannon’s entropy, Approximate Entropy (ApEn), Sample Entropy (SampEn), the fractal dimension using three different algorithms, the Hurst exponent, and the error of the Hurst exponent.
3. Refactor the sub-datasets into different prediction problems, i.e., predicting the consecutive value of 1 previous step, predicting the consecutive value of 2 previous steps, and so on up to 100 previous steps. Thus, we get 100 prediction problems differing in their memory of previous values, i.e., 100 different prediction problems for each sub-interval.
4. Next, we shuffle the data of each sub-interval and spit it into a train and test dataset, with a relative partitioning of 0.8 to 0.2, respectively.
5. We then performed regression analysis using a machine learning algorithm on each prediction problem for each sub-interval and collected the training and test dataset scores.

We performed this procedure first for the regular data and second for the data set that was detrended using the M1 money supply.

### 3.1. Data Sets

We used three data sets for our research: The Dow Jones Industrial Average, Second NASDAQ, and third data on the M1-money supply.

We set our time frame until 31 December 2019 because, after this date, the criteria for the M1-supply changed, i.e., before May 2020, M1 consisted of:

1. Currency outside the U.S. Treasury, Federal Reserve Banks, and the vaults of depository institutions;
2. Demand deposits at commercial banks (excluding those amounts held by depository institutions, the U.S. government, and foreign banks and official institutions), fewer cash items in the process of collection, and Federal Reserve float;
3. Other Checkable Deposits (OCDs), consisting of Negotiable Order of Withdrawal (NOW), and Automatic Transfer Service (ATS), accounts at depository institutions, share draft accounts at credit unions, and demand deposits at thrift institutions.

Beginning with May 2020, the third point changed to other liquid deposits, consisting of OCDs and savings deposits (including money market deposit accounts), which led to an unreasonable increase in the M1 money supply.

#### 3.1.1. M1 Money Supply

- **Time span:** 1 January 1959–1 December 2019;
- **Data:** monthly average;
- **Number of data points:** 732;
- **Source:** [30].

As we have only one value for each month for the M1 money supply data, but several for the other data sets, we used the one available monthly M1-value to make an adjustment for the Dow Jones and NASDAQ data for each day of the corresponding month.

#### 3.1.2. Dow Jones Industrial Average

Dow Jones is a stock market index measuring the performance of 30 large companies listed on stock exchanges in the United States.

- **Time span:** 2 January 1959–31 December 2019;
- **Data:** daily closing values;
- **Number of data points:** 15,359;
- **Source:** [31].

#### 3.1.3. NASDAQ Composite

The NASDAQ Composite is a stock market index that includes almost all stocks listed on the NASDAQ stock exchange.

- **Time span:** 5 February 1971–31 December 2019;
- **Data:** daily closing values;
- **Number of data points:** 12,335;
- **Source:** [32].

### 3.2. Machine Learning Algorithms

We employed three different machine learning algorithms to make predictions on all datasets to ensure the results do not depend on a single regression approach. Thus, we chose XGBoost, a Lasso regression, and a linear stochastic gradient descent regression for our analysis. As we want to keep this article focused, we only briefly mention the referred techniques and give further references for the interested reader.

We chose the employed algorithms because of several reasons. First, tree-based and basic regression algorithms are some of the most common algorithms to perform regression analysis. Further, as a tree-based XGBoost algorithm is conceptionally different from a linear or lasso regression, we expect to capture two main aspects of machine learning algorithms by employing the discussed algorithms. Next, we chose not to employ neural networks because of their inconsistencies in the design. Whereas the employed algorithms are very coherent when it comes to optimizing their design, i.e., see the range of parameters in Section 3.2.4, we cannot think of a way to coherently choose the number of layers and the corresponding neurons with respect to annually changing time-series data and varying complexities, as different design choices may yield completely different results.

#### 3.2.1. Tree Based Extreme Gradient Boosting (XGBoost)

The first algorithm to be presented is an EXtreme Gradient Boosting (XGBoost) tree-based algorithm. In machine learning terminology, the term *boosting* refers to combining the results of many weak predictions to a strong one. Thus the selection of these weak classifiers has to be optimized. Further, boosting is generalizable by allowing optimization of an arbitrary differentiable loss function.

XGboost was proposed in [33] as part of the greedy function approximation. The nowadays standard algorithm was then developed by [34] and is a decision tree-based ensemble method.

We used an existing xgboost=1.2.1 python implementation in combination with sklearn for our research.

#### 3.2.2. Lasso Regression

The Least Absolute Shrinkage and Selection Operator (LASSO) is a regression technique. Part of the LASSO are variable selection and regularization. Both, variable selection and regularization serve to enhance regression analysis to achieve more accurate predictions. The *shrinkage* therefore refers to shrinking data values towards a central point as the mean and is also referred to as *L1* regularization.

The original sources of the LASSO regression are [35,36]. Another interesting read on this method is [37] as it treats LASSO regression in the generalized context of penalized regression models.

We used an existing implementation from sklearn for our analysis.

#### 3.2.3. Stochastic Gradient Descent Linear Regression

We further employed a basic stochastic gradient descent linear regression model from sklearn, i.e., SGDRegressor.

We chose this algorithm as it is one of the most basic machine learning algorithms, and so we can compare more sophisticated results, e.g., from an XGBoost model, to the results of a stochastic gradient descent linear regression model. A stochastic gradient descent linear regression model can be applied to a variety of problems including stock market data  [29,38].

We used an existing implementation from sklearn for our analysis.

#### 3.2.4. Optimization

We further optimized each algorithm by using RandomizedSearchCV from sklearn. We used the following parameters and ranges for optimization:

- **XGBoost:**
      "n_estimators": stats.randint(50, 1200)
      "colsample_bytree": [1, 0.9, 0.8, 0.5, 0.4]
      "eta": stats.expon(scale=.2)
      "max_depth": stats.randint(1, 12)
      "gamma": [0, 2, 4]
      "lambda": stats.uniform(0.0, 2.0)
      "alpha": stats.uniform(0.0, 2.0)
      "min_child_weight": stats.randint(1, 3)}
- **SGDRgressor:**
      "alpha": [1, 0.1, 0.01, 0.001, 0.0001, 0.00001, 0]
      "eta0": [0.1, 0.01, 0.001, 0.0001]
- **Lasso:**
      "alpha": [1, 0.5, 0.25, 0.1, 0.01, 0.001]

### 3.3. Error Metrics

We employed two different error measures and a cross-validation procedure to validate our results:

#### 3.3.1. Root Mean Squared Error (RMSE)

For a signal $\left[x_{1},x_{2},\ldots ,x_{n}\right]$, and a corresponding prediction $\left[{\hat{x}}_{1},{\hat{x}}_{2},\ldots ,{\hat{x}}_{n}\right]$, the root mean squared error (RMSE) is defined as:

(1) $$\mathrm{RMSE}\;=\;\sqrt{\frac{1}{n}\sum_{i=1}^{n}{\left({\hat{x}}_{i}-x_{i}\right)}^{2}}.$$

#### 3.3.2. Coefficient of Determination ($R^{2}$-Score)

We are given a signal $\left[x_{1},x_{2},\ldots ,x_{n}\right]$, and a corresponding prediction $\left[{\hat{x}}_{1},{\hat{x}}_{2},\ldots ,{\hat{x}}_{n}\right]$. We find the mean of the signal as:

(2) $$\bar{x}=\frac{1}{n}\sum_{i=1}^{n}x_{i}.$$

We then calculate the sum of total squares:

(3) $${\mathrm{SS}}_{tot}=\sum_{i}^{n}{\left(x_{i}-\bar{x}\right)}^{2},$$

and the sum of residual squares:

(4) $${\mathrm{SS}}_{res}=\sum_{i}^{n}{\left(x_{i}-{\hat{x}}_{i}\right)}^{2}.$$

Thus we find the coefficient of determination as:

(5) $$R^{2}\;=\;1-\frac{{\mathrm{SS}}_{res}}{{\mathrm{SS}}_{tot}},$$

whereas a value close to 1 is an excellent score, a value close to zero indicates prediction values close to the mean of the actual signal, and a value below zero predictions worse than the baseline of the mean.

#### 3.3.3. Cross Validation

To validate the results and optimize the employed machine learning algorithms, we used an existing implementation for a k-fold cross validation from sklearn with five folds and shuffled data. We did not use a time series-adapted cross-validation procedure for this study. We do not aim to forecast time series data but to make statements on predictability in general. Further, as we studied the data on an annual basis, a time series-adapted cross-validation would have led us to not consider the later months of each year as training data, as time series cross-validation methods are always time-ordered.

### 3.4. Complexity Analysis

We tested several complexity measures on how they relate to the scores of the regression analysis.

#### 3.4.1. Fractal Dimension

The fractal dimension of a time series can be understood as a measure of signal complexity. The basic idea is to first consider the time series as a two-dimensional plot lying on a grid of equal spacing and then count the number of grid boxes necessary to cover the whole time-series data. We thus get a ratio of the overall plot area and the area occupied by the time signal. This process is referred to as *box-counting*. The fractal dimension can have a non-integer value, i.e., the fractal dimension *D* of a self-affine time series can have values 1<D<2.

There are several algorithms to calculate the fractal dimension of a time series, and we used the following three concepts for our research, i.e., the algorithm by Higuchi [39], the algorithm by Petrosian [40], and the algorithm by Katz [41].

#### 3.4.2. Hurst Exponent, R/S Analysis, Hurst-Error

The Hurst exponent measures long-term memory of time series data. It was invented in 1965 and is calculated using R/S analysis [42]. We only use for our research, the necessary excerpt from the theory and refer for to [42,43] for an in-depth treatment of the subject.

*R/S analysis* (Rescaled range analysis) is used to identify long-run correlations in time series. It yields one parameter, the *Hurst exponent* “*H*”.

For a given signal $\left[x_{1},x_{2},\ldots ,x_{n}\right]$, we find the average over a period τ (a sub-interval of the signal, i.e., 1≤τ≤n), with *k* as 1≤k≤n and elements *i* in this interval such that k≤i≤k+τ:

(6) $${\langle x\rangle }_{\tau ,k}\;=\;\frac{1}{\tau }\sum_{j=k}^{k+\tau }\;x_{j}.$$

Further, we find the *accumulated departure* $\delta x\left(i,\tau ,k\right)$ over period a period i∈1,2,…,τ as:

(7) $$\delta x\left(i,\tau ,k\right)\;=\;\sum_{j=k}^{i}\left(x_{j}-{\langle x\rangle }_{\tau ,k}\right).$$

Next we find the range *R*, which is the the difference between maximal and minimal values of all $x_{i}$ in the interval $\left[k,k+\tau \right]$ as:

(8) $$\begin{array}{cc} R\left(\tau ,k\right)\; & =\;\max\left[\delta x\left(i,\tau ,k\right)\right]\;-\;\min\left[\delta x\left(i,\tau ,k\right)\right]\;,\\ \; & \mathrm{satisfying}\;\;k\leq i\leq k+\tau \;. \end{array}$$

The corresponding standard deviation for each subinterval is:

(9) $$S\left(\tau ,k\right)\;=\;\sqrt{\frac{1}{\tau }\sum_{i=k}^{k+\tau }{\left[x_{i}-{\langle x\rangle }_{\tau ,k}\right]}^{2}}.$$

For the final range and the standard deviation, we average our previous findings over all possible (The algorithms that perform R/S analysis find a subset of possible intervals and do perform the procedure on all possible intervals.) *k* as:

(10) $$\begin{array}{cc} \; & R\left(\tau \right)\;=\;\frac{\sum_{k}R\left(\tau ,k\right)}{\mathrm{number}\mathrm{of}\mathrm{different}ks}\\ \; & \;\mathrm{and}\\ \; & S\left(\tau \right)\;=\;\frac{\sum_{k}S\left(\tau ,k\right)}{\mathrm{number}\mathrm{of}\mathrm{different}ks}, \end{array}$$

where 1≤k≤n and k≤i≤k+τ. The *Hurst exponent* *H* is then defined using the scaling properties as:

(11) $$\frac{R\left(\tau \right)}{S\left(\tau \right)}\;\propto \;\tau^{H}.$$

The asymptotic behavior for an independent random process with finite variance is then given as:

(12) $$\frac{R\left(\tau \right)}{S\left(\tau \right)}\;=\;{\left(\frac{\pi }{2v}\tau \right)}^{\frac{1}{2}},$$

thus implying $H=\frac{1}{2}$ for random processes. For real-life data, $H\neq \frac{1}{2}$, as most real-life processes feature long-term correlations.

The range of *H* is 0<H<1. A value H<0.5 indicates anti-persistency, i.e., heavily fluctuating, however not completely random. Values close to 0 are characteristic of strong anti-persistency. On the other side, H>0.5, which indicates persistent behavior, thus strong persistency for values close to 1. Further, time-series H≠0.5 can theoretically be forecast, [12].

For R/S analysis, we used the python packages https://pypi.org/project/nolds/, ref. [44], and https://github.com/Mottl/hurst, accessed on 17 January 2022.

**Figure 1:**
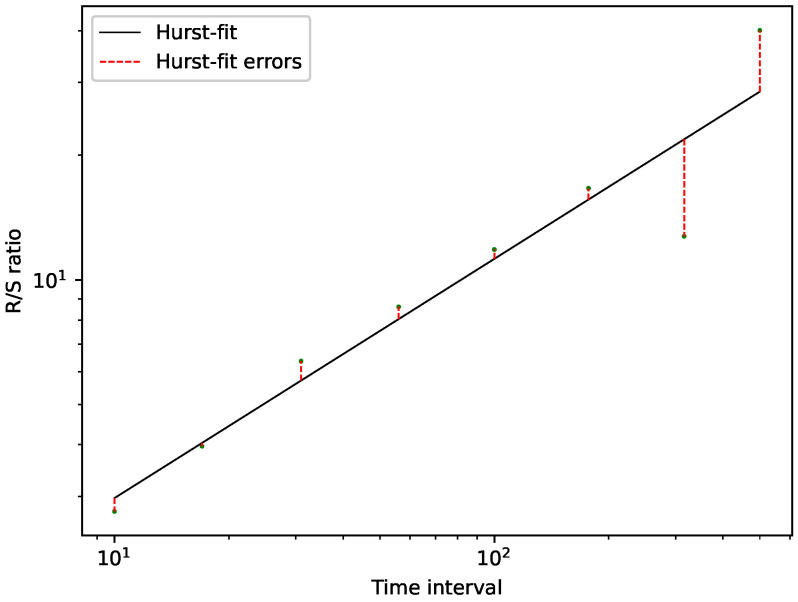
Double logarithmic plot for the fit of the Hurst exponent for a random walk with a probabilty of 0.5 and a length of 500 steps. The calculated Hurst exponent is H=0.57, and the corresponding Hurst-error is ${\mathrm{RMSE}}_{\mathrm{Hurst}}=5.229$. This results from different, i.e., larger, fluctuations for larger time intervals than for smaller time intervals.

To visualize R/S analysis, we plot the ratio on a logarithmic scale against the intervals, also on a logarithmic scale. Thus the Hurst exponent is the slope of the corresponding linear fit, see Figure 1.

We can then find a new parameter related to R/S analysis by measuring the distance of the actual data points to the Hurst-fit, i.e., the linear fit of the double logarithmic scale. We measure this distance, i.e., the residuals, using a root mean squared error. Throughout this research, we will refer to this error of the Hurst-fit as *Hurst-error*. The importance of this Hurst-error is its ability to differentiate between mono-fractal or multi-fractal time series data. If we are thus given two-time series with the same Hurst exponent, and we find a difference between their Hurst errors, we can state that the time series with the larger Hurst error is a more multi-fractal one, i.e., the fluctuations differ on different scales. On the other hand, if we find a time series with a Hurst-error of zero, we can state that this is a perfectly mono-fractal time series, meaning that we find very similar fluctuations on all scales. The Hurst-fit and the corresponding Hurst-error are visualized in Figure 1 (Note that usually, one would not get such large deviations from the fit for a fractional Brownian motion, but we altered this test-data such that the plot is explanatory and indicative).

#### 3.4.3. Fisher’s Information

Fisher’s information is the amount of information extracted from a set of measurements, i.e., the quality of the measurements [45]. It can be interpreted as a measure of order or disorder of a system or data, thus it can be used to investigate non-stationary and complex signals.

Fisher’s information is suitable for univariate time series analysis, given as a signal $\left[x_{1},x_{2},\ldots ,x_{n}\right]$.

First, we construct embedding vectors as:

(13) $$\vec{y}\left(i\right)\;=\;\left[x_{i},x_{i+\tau },\ldots ,x_{i+\left(d_{E}-1\right)*\tau }\right],$$

with time delay τ and an embedding dimension $d_{E}$. The embedding space, as a matrix, then is:

(14) $$Y\;=\;{\left[\vec{y}\left(1\right),\vec{y}\left(2\right),\ldots ,\vec{y}\left(N-\left(d_{E}-1\right)\tau \right)\right]}^{T}.$$

Next, we perform a single value decomposition, [46], yielding *M* singular values $\sigma_{i}$ with the corresponding normalized singular values:

(15) $${\bar{\sigma }}_{i}\;=\;\frac{\sigma_{i}}{\sum_{j=1}^{M}\sigma_{j}}.$$

Fisher’s information is then:

(16) $$I_{\mathrm{Fisher}}\;=\;\sum_{i=1}^{M-1}\frac{{\left[{\bar{\sigma }}_{i+1}-{\bar{\sigma }}_{i}\right]}^{2}}{{\bar{\sigma }}_{i}}.$$

Here the implementation from the python package https://neurokit.readthedocs.io/en/latest/, (accessed on 17 January 2022) [47], was used. This implementation requires two parameters, first the time delay, which was found using the calculation of the average mutual information from [48] and the embedding dimension, which was determined using a false nearest neighbor algorithm [49]. The results for both the embedding dimension and the time delay are depicted in Appendix C.

#### 3.4.4. Approximate Entropy (*ApEn*)

Developed by Steve M. Pincus, *approximate entropy* was originally used to analyze medical data [19] with applications to general biologic network systems in later works [50].

We used the python package https://github.com/raphaelvallat/antropy, (accessed on 17 January 2022) to calculate the approximate entropy of a data set.

*ApEn* assigns a non-negative number to a time series, where bigger values indicate greater randomness rather than smaller values. Further, *ApEn* can be seen as an ensemble parameter of process auto-correlation, i.e., smaller values correspond to greater positive auto-correlation, and larger values indicate greater independence.

Given a signal $\left[x_{1},x_{2},\ldots ,x_{n}\right]$, we first fix two input parameters *m* and *r*, where *m* is the length of compared runs, i.e., the embedding dimension, and *r* is a necessary filter parameter. The embedding dimension was determined using a false nearest neighbor algorithm [49]. The results for the embedding dimension are depicted in Appendix C. We then take subsets to form vector sequences ${\vec{x}}_{i}=\left[x_{i},x_{i+1},\ldots x_{i+m-1}\right]$, whereas i+m−1≤n. The vectors ${\vec{x}}_{i}$ therefore represent *m* consecutive values *x* of the signal, addressed to as the *i*th point of the signal.

Next, we define a distance $d\left[{\vec{x}}_{i},{\vec{x}}_{j}\right]$ between vectors ${\vec{x}}_{i}$ and ${\vec{x}}_{j}$ as the maximum difference in their respective scalar components.

We then measure the regularity and frequency of patterns within a tolerance *r* as:

(17) $$C_{i}^{m}\left(r\right)\;=\;\frac{\mathrm{number}\mathrm{of}{\vec{x}}_{j}\mathrm{such}\mathrm{that}d\left[{\vec{x}}_{i},{\vec{x}}_{j}\right]\leq r}{n-m+1}.$$

Next, we define:

(18) $$\Phi^{m}\left(r\right)\;=\;\frac{1}{n-m+1}\sum_{i=1}^{n-m+1}\log C_{i}^{m}\left(r\right),$$

where log is the natural logarithm. Approximate entropy is then found as:

(19) $$\mathrm{ApEn}\left(m,r,N\right)\;=\;\Phi^{m}\left(r\right)\;-\;\Phi^{m+1}\left(r\right).$$

*ApEn* can be interpreted as a likelihood of similar patterns of observations to not be followed by additional similar observations, i.e., a time series containing repetitive regular patterns has a lower *ApEn* value than a more irregular time series. *ApEn* thus evaluates both dominant and subordinate patterns in data, reflecting irregularities on all scales.

#### 3.4.5. Sample Entropy (*SampEn*)

Given a signal $\left[x_{1},x_{2},\ldots ,x_{n}\right]$, we again find an embedding dimension *m* and a filter value *r*. The embedding dimension was determined using a false nearest neighbor algorithm, [49]. The results for the embedding dimension are depicted in Appendix C. We then take subsets to form vector sequences ${\vec{x}}_{i,m}=\left[x_{i},x_{i+1},\ldots x_{i+m-1}\right]$, whereas i+m≤n. $\mathrm{SampEn}\left(m,r,n\right)$ is then the negative value of the logarithm of the conditional probability that two similar sequences of *m* points remain similar at the next point m+1, i.e., the embedding dimension is increased by 1, thus counting each vector over all the other vectors except on itself [51]. Therefore *SampEn* maintains the relative consistency and is also mostly independent of the length of the series.

Though similar, *SampEn* has some subtle differences compared to *ApEn*. For *SampEn*, the time series is used as a whole, thus requiring a template vector to find a match of length m+1 to be defined. For *ApEn*, each template vector has to find a match to be defined.

To get *SampEn*, we first calculate two coefficients $A^{m}\left(r\right)$ and $B^{m}\left(r\right)$:

(20) $$\begin{array}{cc} A_{i,n}^{m}\left(r\right) & =\frac{1}{n-m-1}\;\sum_{j=1,j\neq i}^{n-m}\left(\mathrm{number}\mathrm{of}\mathrm{times}\mathrm{that}d\left(\left|{\vec{x}}_{j,m+1}-{\vec{x}}_{i,m+1}\right|\right)<r\right)\;\mathrm{and}\\ B_{i,n}^{m}\left(r\right) & =\frac{1}{n-m-1}\;\sum_{j=1,j\neq i}^{n-m}\left(\mathrm{number}\mathrm{of}\mathrm{times}\mathrm{that}d\left(\left|{\vec{x}}_{j,m}-{\vec{x}}_{i,m}\right|\right)<r\right). \end{array}$$

Summing over *i*, thus yields:

(21) $$\begin{array}{cc} A_{n}^{m}\left(r\right) & =\frac{1}{n-m}\sum_{i=1}^{n-m}A_{i,n}^{m}\left(r\right)\;\mathrm{and}\\ B_{n}^{m}\left(r\right) & =\frac{1}{n-m}\sum_{i=1}^{n-m}B_{i,n}^{m}\left(r\right). \end{array}$$

The statistic sample entropy is then defined as:

(22) $$\mathrm{SampEn}\left(m,r,n\right)\;=\;-\log\left[\frac{A_{n}^{m}\left(r\right)}{B_{n}^{m}\left(r\right)}\right].$$

Here log is the logarithm with base *e*, i.e., Euler’s number. From this, the sample entropy can be estimated as:

(23) $$\mathrm{SampEn}\left(m,r\right)\;=\;\lim_{n\to \infty }\left(-\log\left[\frac{A_{n}^{m}\left(r\right)}{B_{n}^{m}\left(r\right)}\right]\right).$$

A larger value of SampEn indicates a more complex time series data, whereas a smaller value of SampEn indicates a more regular and self correlated time series data.

We only covered the basic ideas of both ApEn and SampEn and the interested reader is referred to [51,52] for an in-depth treatment of the subject.

We used the python package https://github.com/raphaelvallat/antropy, (accessed on 17 January 2022) to calculate the approximate entropy of a data set.

#### 3.4.6. Shannon’s Entropy

Given a signal $\left[x_{1},x_{2},\ldots ,x_{n}\right]$, the probability for each value to occur is $P\left(x_{1}\right),\ldots ,P\left(x_{n}\right)$, thus we denote Shannon’s entropy, [53], as:

(24) $$H_{\mathrm{Shannon}}\;=\;-\sum_{i=1}^{n}P\left(x_{i}\right)\log_{2}P\left(x_{i}\right).$$

As the base of the logarithm is set to 2, we refer to its content as bits. Some applications are: Astronomy [54], to identify periodic variability; or in finance, [55] to measure and estimate risks for investments. Shannon’s entropy basically measures the uncertainty of processes/signals.

### 3.5. M1 Money Supply Detrending

We performed every described technique on two versions of a data set, first, the original data set, and second, a data set that was detrended with the M1 money supply.

For our M1 detrending, we used a data set from https://fred.stlouisfed.org, accessed on 17 January 2022. We used monthly data from 1959 to 2019. Thus, we set the monthly value constant for each day of a month. Next, given our M1 data set as a signal $\left[x_{1},x_{2},\ldots x_{n}\right]$, we divided the whole data set by the first value $x_{1}$, to get a normalized data set, describing the relative changes of the M1 money supply with respect to the first value, i.e.,:

(25) $$\left[x_{1},x_{2},\ldots x_{n}\right]\;\to \;\left[\frac{x_{1}}{x_{1}},\frac{x_{2}}{x_{1}},\ldots \frac{x_{n}}{x_{1}}\right]\;:=\;\left[{\hat{x}}_{1},{\hat{x}}_{2},\ldots {\hat{x}}_{n}\right].$$

Given our stock market data as a signal $\left[y_{1},y_{2},\ldots y_{n}\right]$, we then divided by our normalized data set, to get the detrended dataset, denoted as $\left[{\hat{y}}_{1},{\hat{y}}_{2},\ldots {\hat{y}}_{n}\right]$, i.e.,:

(26) $$\left[y_{1},y_{2},\ldots y_{n}\right]\;\to \;\left[\frac{y_{1}}{{\hat{x}}_{1}},\frac{y_{2}}{{\hat{x}}_{2}},\ldots \frac{y_{n}}{{\hat{x}}_{n}}\right]\;:=\;\left[{\hat{y}}_{1},{\hat{y}}_{2},\ldots {\hat{y}}_{n}\right].$$

And third, we can find a signal describing the relative change of the M1 money supply with respect to the stock market data, thus:

(27) $$\left[{\hat{x}}_{1},{\hat{x}}_{2},\ldots {\hat{x}}_{n}\right]\;\to \;\left[{\hat{x}}_{1}\times y_{1},{\hat{x}}_{2}\times y_{1},\ldots {\hat{x}}_{n}\times y_{1}\right]\;:=\;\left[z_{1},z_{2},\ldots z_{n}\right].$$

**Figure 2:**
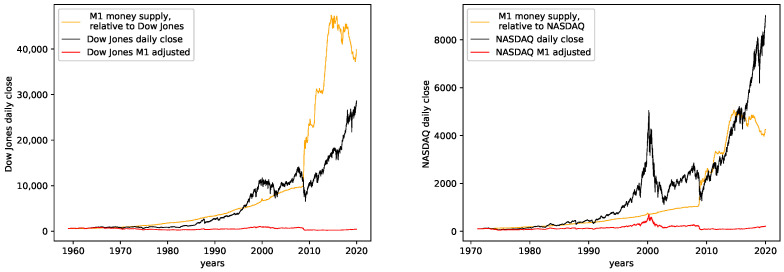
M1 detrended and relative plots for both the Dow Jones and the Nasdaq daily close data. The yellow data therefore are the signal $\left[z_{1},z_{2},\ldots z_{n}\right]$, the black data are the signal $\left[y_{1},y_{2},\ldots y_{n}\right]$, and the red data are the signal $\left[{\hat{y}}_{1},{\hat{y}}_{2},\ldots {\hat{y}}_{n}\right]$.

The corresponding plots for both data sets can be found in Figure 2.

## 4. Results and Discussion

In this section, we show all the results from the tools discussed in Section 3, for all mentioned data sets. We further discuss the results using Pearson’s correlation coefficient [56], the $\chi^{2}$-test [57], and interpret them qualitatively.

### 4.1. Complexity Analysis

**Figure 3:**
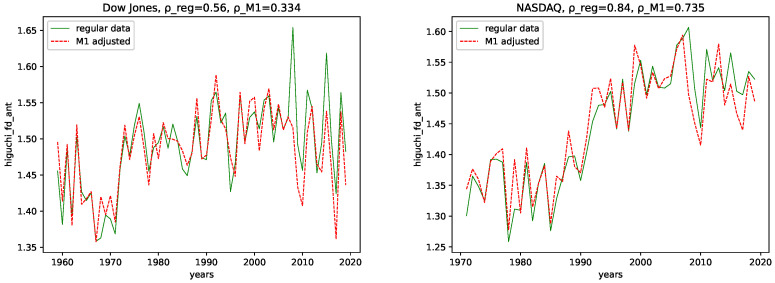
Fractal dimension calculated using the algorithm by Higuchi plotted for each year and for both the M1 money supply data and the regular data.

**Figure 4:**
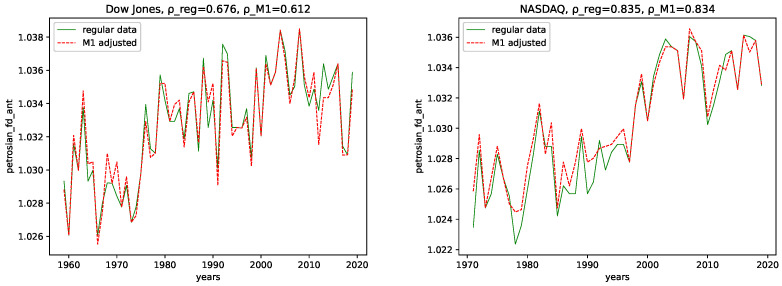
Fractal dimension calculated using the algorithm by Petrosian plotted for each year and for both the M1 money supply data and the regular data.

**Figure 5:**
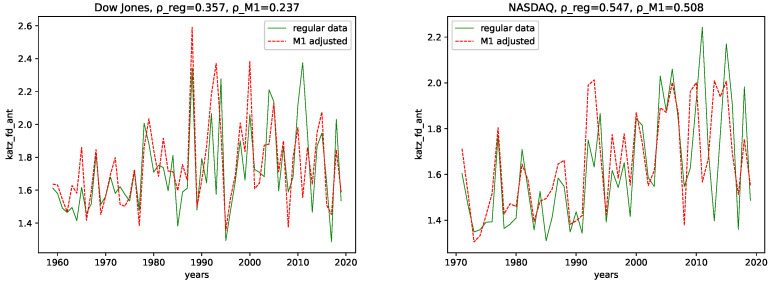
Fractal dimension calculated using the algorithm by Katz, plotted for each year and for both the M1 money supply data and the regular data.

**Figure 6:**
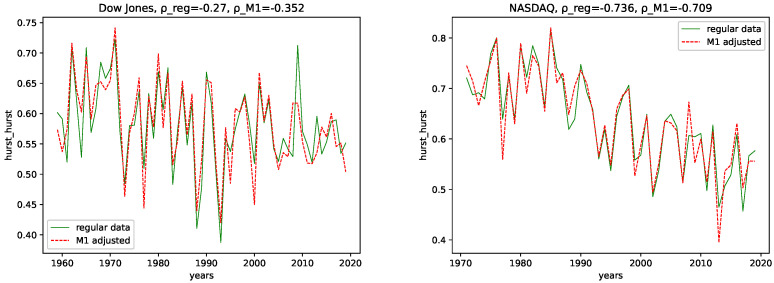
Hurst exponent plotted for each year and for both the M1 money supply data and the regular data.

**Figure 7:**
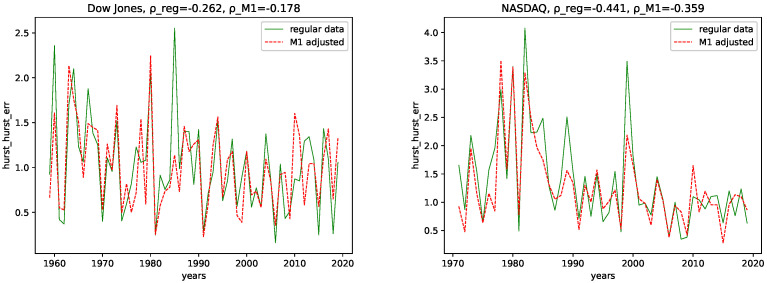
Hurst-error plotted for each year and for both the M1 money supply data and the regular data.

**Figure 8:**
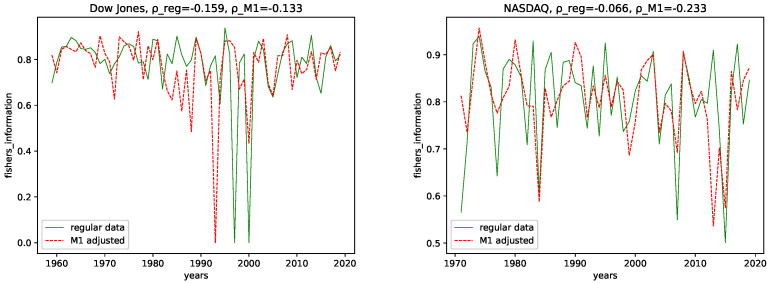
Fisher’s information plotted for each year and for both the M1 money supply data and the regular data.

**Figure 9:**
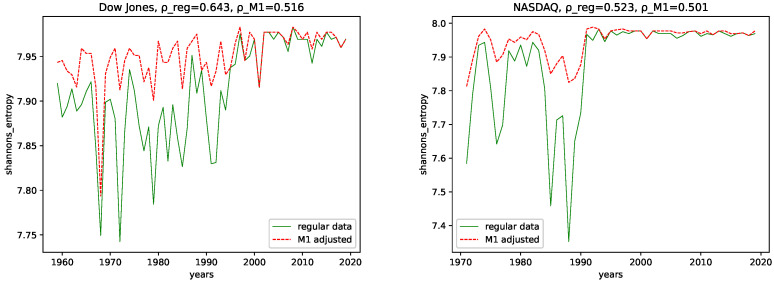
Shannon’s entropy plotted for each year and for both the M1 money supply data and the regular data.

**Figure 10:**
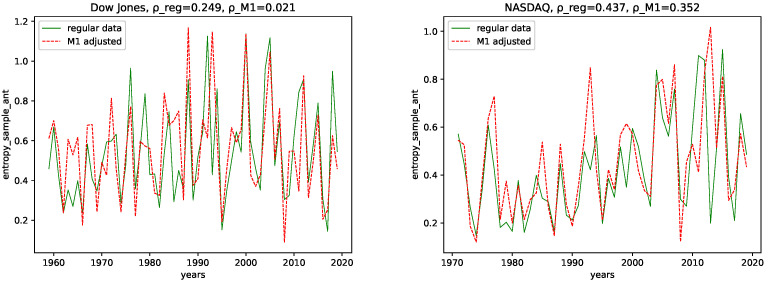
Sample entropy plotted for each year and for both the M1 money supply data and the regular data.

**Figure 11:**
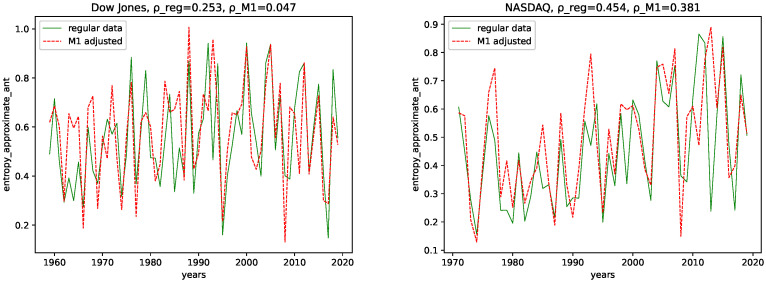
Approximate entropy plotted for each year and for both the M1 money supply data and the regular data.

We calculated all the complexity measures mentioned and discussed in Section 3.4 for each year and each data set, for both the M1-detrended data and the regular data. Further, we give its estimated correlation using Pearson’s correlation coefficient in each plot, where ρ_reg is the coefficient for the regular data, and ρ_M1 is the coefficient for the M1-detrended data. The results can be seen in the plots Figure 3, Figure 4, Figure 5, Figure 6, Figure 7, Figure 8, Figure 9, Figure 10 and Figure 11.

Next, we discuss the results of these plots qualitatively for their implications.

#### 4.1.1. Comparison: M1-Detrended vs. Non-Detrended Data

First, we discuss the differences in the signal complexities for the M1-detrended vs. the non-detrended data.

**Fractal dimension:** We observe very similar behavior for both the M1-detrended and the non-detrended data for all three of the employed measures for the fractal dimension for both data sets.

The correlation coefficient has the same sign for both data sets and all fractal dimensions and suggests a positive correlation. Further, the correlation coefficient is always larger for the non-detrended data. This indicates that the M1-detrending adds additional disorder/noise to the data in this case.

**Hurst exponent and Hurst-error:** We observe a negative correlation for both data sets and the M1-detrended and non-detrended data for the Hurst exponent. Here, the correlation coefficient for the Dow Jones data for the regular data has a lower value than the M1-detrended data, whereas it is the opposite for the NASDAQ data. Thus, we conclude that the M1 detrending adds noise to the Dow Jones data, reducing the disorder for the NASDAQ data. We see some differences for the Hurst-error, i.e., the non-detrended data is more expressive with higher peaks and larger correlation coefficients for both data sets.

**Fisher’s Information:** We see a similar behavior as that for the Hurst-error for Fisher’s information. Though the regular data seems to be more expressive in general, we see a difference in some significant low peaks for the Dow Jones. Whereas Fisher’s information shows two low peaks around 2000 for the regular data, we find only one for 1993 for the M1-detrended data. Further, we observe that many smaller low peaks of the NASDAQ data are flattened by the M1 adjustment. Thus we conclude, as Fisher’s information is, just as the name says, a measure for information and that M1-adjustment reduces the inherent information for some intervals and adds some information for others. Our interpretation for this behavior is: The M1 money supply has a weak influence on the stock understudy for intervals where M1-adjustment adds information. The values for the correlation coefficients for Fisher’s information for both data sets are very low, i.e., below 0.25 (This is not an empirical threshold, but as the plots suggest, there is no apparent trend. Thus we focus on the difference in the peaks); we do not include it in this comparison.

**Shannon’s entropy:** We observe the most significant changes of behavior for Shannon’s entropy. Here the regular data contains less entropy for older data for both data sets. For more recent data, starting with ≈1993 for NASDAQ and ≈1998 for Dow Jones, we observe very similar behavior for both the M1-detrended and the non-detrended data. We interpret this behavior similarly to Fisher’s information. Thus, the M1 money supply has a weak influence on the intervals where M1-adjustment increases the entropy as it adds additional disorder. However, the influence becomes stronger with a diminishing difference in the entropy. Given the actual charts of the data sets and the M1 money supply, Figure 2, we see that both stock market data and the M1 money supply show stronger fluctuating (and steep increasing) behavior starting with the mid-90s, which is approximately the time where we propose for the M1 money supply to have a stronger influence on the stock market data under study.

We can also observe this behavior in the correlation coefficients, i.e., the correlation coefficient for the non-detrended data has a larger value for both data sets, i.e., the M1 adjustment adds noise to the data sets.

**ApEn and SampEn:** Though there are some differences when considering the M1-detrended and the non-detrended data, we do not observe an obvious pattern to be discussed here. We can also see that the correlation coefficient is always larger for the non-detrended data. Thus, we conclude that the M1 adjustment adds noise to the data, i.e., the regular data is more expressive than the M1-detrended one.

#### 4.1.2. Temporal Behavior

Next, we discuss how the signal complexity changes with the years.

**Fractal dimensions and Hurst exponent:** We observe changing behavior over time for all three employed fractal dimension measures and the Hurst exponent. Given the relation from [58] that relates the Hurst exponent and the fractal dimension of a time series, i.e., 2−H≈FD (Which seems to hold only approximately for Higuchi’s fractal dimension), where *H* is the Hurst exponent, and FD is the fractal dimension, we interpret the findings of these trends of using both simultaneously, the Fractal dimension and the Hurst exponent. Given the fractal dimension and the behavior of the Hurst exponent for both the M1-detrended and non-detrended NASDAQ data, we observe an increase in signal complexity, i.e., an increasing Fractal dimension for later years. Moreover, just as the Fractal dimension increases, we observe the decreasing behavior of the Hurst exponent towards a value of ≈0.5, which indicates a random process. Thus, we conclude that the NASDAQ stock index, as a process, became more random for later years, and we propose for future years for NASDAQ to fluctuate around a Hurst exponent of 0.5, because this value is the limit as it indicates maximal radomness.

We can also observe an increasing Fractal dimension for the Dow Jones, at least for the Higuchi’s and Petrosian’s fractal dimensions. Though it is not as distinct as for the NASDAQ data. The Hurst exponent for the Dow Jones index seems to fluctuate around (or slightly above) 0.5 for most years. Thus, we conclude that the Dow Jones Stock market index is, as a process, inherently more random than the NASDAQ index.

All fractal dimension measures show a positive correlation coefficient for all data, indicating increasing signal complexity. Further, the correlation coefficient value is always larger for the NASDAQ data, which indicates a stronger trend for NASDAQ than for Dow Jones. For both data sets and for both the M1 detrended data and the non-detrended data, we observe negative correlation coefficients for the Hurst exponent. Moreover, as this indicates that the Hurst exponent converges towards 0.5, it means increasing randomness for later years.

**The Hurst-error:** The to be discussed behavior for the Hurst-error for both NASDAQ and Dow Jones is very similar for both the M1-detrended and non-detrended data. We, therefore, refer to both the M1-detrended and non-detrended data for the discussion of the Hurst-error.

The Hurst-error for NASDAQ shows smaller fluctuations for later years. Moreover, though the Dow Jones index shows smaller fluctuations for more recent years, this trend is not as distinct as for NASDAQ. However, given the range of the Hurst-error for the Dow Jones, it ends up, around 2010, just as NASDAQ, around 1, with maxima up to 1.5. We conclude that a smaller Hurst error indicates a more random process when interpreting these trends. This originates from the fact that we can build random walks using the Hurst exponent, i.e., the Hurst exponent controls the probability of a random process to change direction, which is called a fractional Brownian motion [59]. Thus, a Hurst exponent of 0.5 gives an equal probability of changing or not changing the direction of the random walk, in our case, an increase or decrease. Given that, the smaller the error of Hurst’s power law to determine the Hurst exponent, the closer the process understudy is to an actual random walk or a fractional Brownian motion. This means that multifractal behavior, i.e., large Hurst errors, i.e., different magnitudes of fluctuations on different scales, are distinctive for non-random-walk processes. On the contrary, small Hurst errors indicate that the observed behavior is closer to an actual random walk. Thus, both NASDAQ and Dow Jones seem to converge towards a random process for later years.

The correlation coefficient also shows this behavior, as, for both NASDAQ and Dow Jones, the coefficients are negative for both the M1-detrended and the non-detrended data.

**Shannon’s entropy:** Shannon’s entropy indicates increasing disorder over the years for both NASDAQ and Dow Jones and both the M1-detrended and non-detrended data.

**ApEn and SampEn:** For NASDAQ, we observe for both ApEn and SampEn and both the M1-detrended and the non-detrended, an increase in the range of the fluctuations, with the overall maxima in the most recent 10 years. We thus conclude that the inherent disorder in the NASDAQ data increased over the years. We can only state that we observe smaller fluctuations until the 1990s for both ApEn and SampEn and both the M1-detrended and the non-detrended data for the Dow Jones index. For later years, we observe an increase in the range of fluctuations, however it is not as evident as for NASDAQ.

In addition, the correlation coefficients support this assumption, as they are positive for all data sets. Thus, we conclude that we are dealing with data with an increasing disorder for later years in terms of Shannon’s entropy.

### 4.2. Machine Learning Predictability

We tested the predictability of machine learning algorithms using an XGBoost, a Lasso, and an SGD regressor. For the error metrics, we employed an R2 5-fold cross-validation score (denoted as R2CV) on the training data, an R2 score (denoted as R2) on the test data, and a root mean squared error (denoted as RMSE) on the test data.

As described in Section 3, we did 100 runs with a varying memory of the algorithms for each data for each year, i.e., an algorithm that looks back 1,2,3…,100 steps into the past, to predict one step into the future. From these 100 predictions for each year, we picked only the results with maximal R2CV and R2 scores and minimal RMSE. The plots for the corresponding memories can be found in Appendix A.

**Figure 12:**
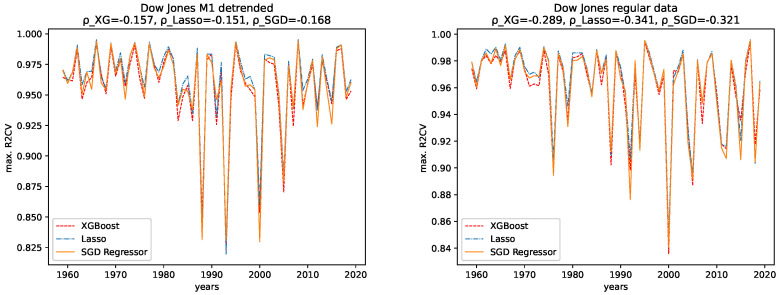
Maximal R2 cross validation score on the regular and the M1-adjusted Dow Jones data for all regressors.

**Figure 13:**
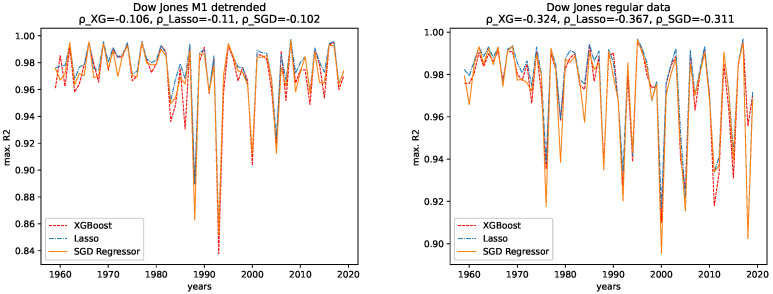
Maximal R2 test data score on the regular and the M1-adjusted Dow Jones data for all regressors.

**Figure 14:**
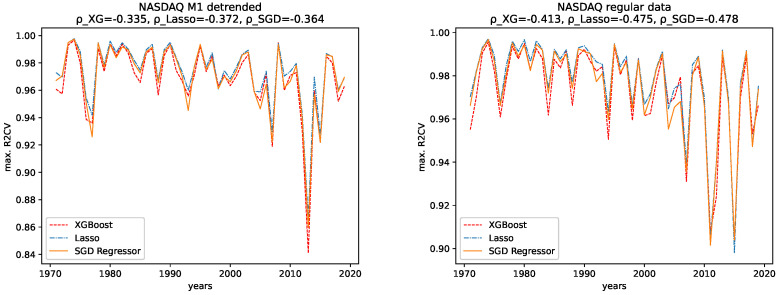
Maximal R2 cross validation score on the regular and the M1-adjusted NASDAQ data for all regressors.

**Figure 15:**
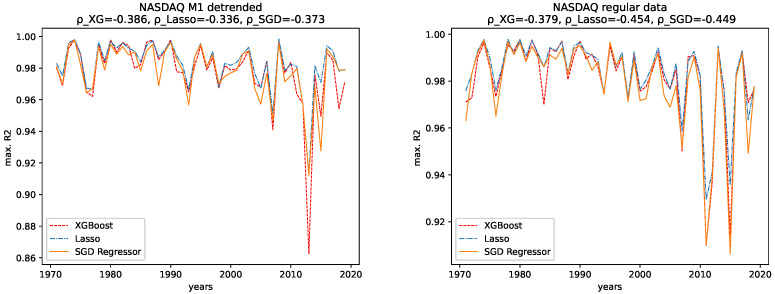
Maximal R2 test data score on the regular and the M1-adjusted NASDAQ data for all regressors.

**Figure 16:**
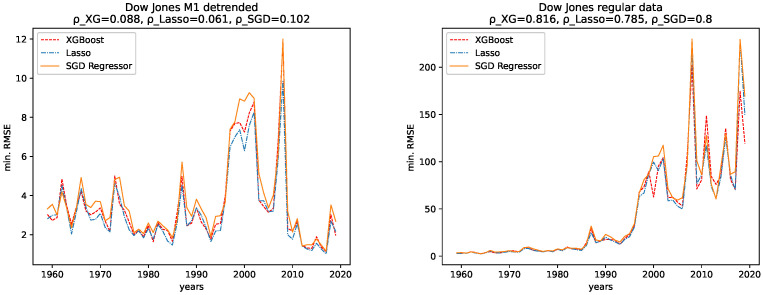
Minimal RMSE on the test data set fort the regular and the M1-adjusted Dow Jones data for all regressors.

**Figure 17:**
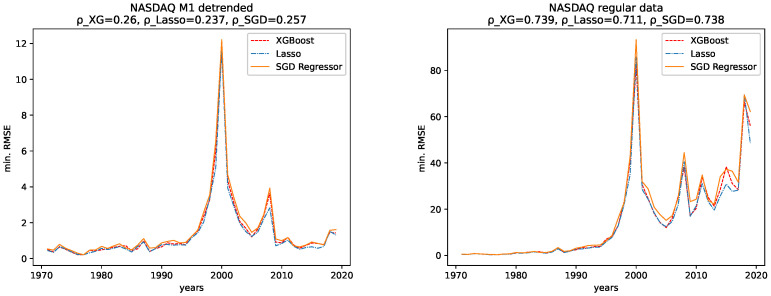
Minimal RMSE on the test data set fort the regular and the M1-adjusted NASDAQ data for all regressors.

The results for both data sets, NASDAQ and Dow Jones, and both the M1 detrended and the regular data are shown, together with the corresponding correlation coefficients (i.e., Spearman’s rank correlation coefficient [56]) in Figure 12, Figure 13, Figure 14 and Figure 15 for the R2CV and R2 scores. For the RMSE analysis, the results are shown in the Figure 16 and Figure 17. Here, ρ_XG is the correlation coefficient for the results for XGBoost, ρ_Lasso for the Lasso regressor, and ρ_SGD for the linear stochastic gradient descent regressor.

**Figure 18:**
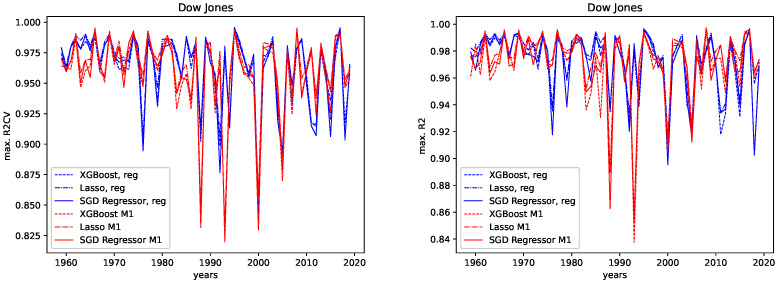
Maximal R2 cross validation and R2 score on the regular and the M1-adjusted Dow Jones data for all regressors.

**Figure 19:**
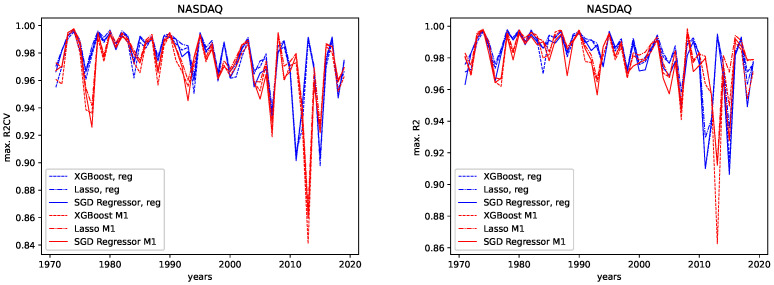
Maximal R2 cross validation and R2 score on the regular and the M1-adjusted NASDAQ data for all regressors.

We further give the errors/scores averaged over all years and the corresponding standard error in Table 1 and Table 2. We also show the results for the M1-detrended and thew non-detrended data in Figure 18 and Figure 19.

#### 4.2.1. Comparison: XGBoost vs. Lasso Regression vs. SGD Linear Regression

We observe the same pattern for both data sets, Dow Jones and NASDAQ, and subsequently for both the M1-detrended and the non-detrended data. Overall, the Lasso performs best on all data, followed by XGBoost, which performs slightly worse. The linear SGD regression gives the worst performance on all data.

#### 4.2.2. Comparison: M1-Detrended vs. Non-Detrended Data

We now take a close look at how the results for the M1-detrended data differs from the results obtained from the non-detrended data for each measure of signal complexity.

**Dow Jones:** For the Dow Jones data, we see that we observe a strong low peak in the year 2000, which is present in the R2CV score for both the M1 detrended and the non-detrended data. We can also see this low peak for the R2 scores on unknown data. The other low peaks in the 1980s and 1990s vary from R2CV to R2 scores and from M1-detrended to non-detrended data. Overall, we observe the highest R2CV scores for both the M1-detrended and the non-detrended data in the 1960s. Afterward, the R2CV score plummets for the non-detrended data. For the M1-detrended data, we observe this decrease in the R2CV starting with the 1980s. We observe similar behavior for the R2 score on unknown data. Interestingly, we observe increasing R2 and R2CV score fluctuations with a relatively small range and higher scores starting around 2010. This behavior is not present for the non-detrended data.

Furthermore, given the results of the correlation coefficients, a trend towards lower predictability for later years is more obvious for the non-detrended data. This holds for all three error measures, R2CV, R2, and RMSE. Whereas the increasing values of the data cause the solid trend for the RMSE.

We further observe, on average, a better predictability score for the regular data than for the M1-detrended data. However, taking a look at the large errors and Figure 18, we see that the M1 detrending improved the predictability for some regions and worsened it for others.

**NASDAQ:** For the Nasdaq data, we observe an extreme low peak of both the R2CV and the R2 score in 2013. This low peak is not present in the regular data. Instead, we observe two low peaks in 2011 and 2015. Besides that, both the M1-detrended and the regular data show similar behavior when it comes to increasing fluctuations of the predictability, which is most evident for the regular data. Thus we conclude, that M1-adjustment did change the predictability for NASDAQ locally. However, the overall pattern to observe slightly lower scores (and course prominent low peaks) for later years is present in both the M1-detrended and the non-detrended data.

In addition, given the results of the correlation coefficients, a trend towards lower predictability for later years is more obvious for the non-detrended data. This holds for all three error measures, R2CV, R2, and RMSE, and all algorithms. Whereas the increasing values of the data cause a strong trend for the RMSE.

We further observe, on average, a better predictability score for the regular data than for the M1-detrended data. However, taking a look at the large errors and Figure 19, we see that the M1 detrending improved the predictability for some regions and worsened it for others.

#### 4.2.3. Temporal Behavior

Now we discuss how the predictability changes from earlier years to later years. **Dow Jones:** For the M1-detrended Dow Jones data, we observe good performance for early years, an increase in the fluctuations and a corresponding decrease of the R2CV and R2 scores, and finally, another increase of these scores and diminished fluctuations of the scores for the later years, starting around 2010. For the non-detrended Dow Jones data, we also observe high initial scores and corresponding fluctuations with a small range, however, afterward, a decrease of the scores and correspondingly, bigger fluctuations are found. When analyzing the RMSE on unknown data, we observe exploding errors for later years for the non-detrended data, which is due to the overall larger values of the data set in this regime; see Figure 2. For the M1-detrended data, we observe high error peaks in the interval 1995–2010 and lower values afterward. If we take a closer look at Figure 2, we see that these peaks, and especially the lower errors, in the end, are caused by the varying value range of the data. Thus we can not interpret the RMSE for the Dow Jones data with respect to its predictability.

When it comes to the corresponding correlation coefficients, we observe negative correlation coefficients, even if some are very low, and are thus not very expressive, for all algorithms, both the M1-detrended and the non-detrended data and both error measures. Thus, we conclude that the predictability tends to be lower for more recent years. We further observe positive correlation coefficients for the RMSE analysis on both the detrended and the non-detrended data. Here, though the non-detrended analysis has a strong positive correlation due to increasing data values, we also conclude this to indicate lower predictability for later years.

**NASDAQ:** For the regular data, we observe an increasing range of the fluctuations and lower values for the R2CV and the R2 score on unknown data for later years. We observe similar behavior in the M1-detrended data, however it is not as evident as for the non-detrended data, despite one strong low peak in 2013. The RMSE for later years for the non-detrended data also increases, as does its value range, so the increasing RMSE for the regular data is not much of use. For the M1-detrended data, we observe transitions, such as the one in 2009, visible in the RMSE. Apart from that, the RMSE is bound to the variation in the value range of the data under study.

When considering the corresponding correlation coefficients, we observe negative values for both error measures, i.e., R2CV and R2, for both data sets, i.e., M1-detrended and the non-detrended data. Thus we conclude that the predictability tends to be lower for more recent years. Regarding the RMSE analysis, we see a prominent peak for the M1-detrended data in 2000, which distorts the analysis. However, still, we observe positive correlation coefficients for both the M1-detrended and the non-detrended data. Though the exploding errors for the non-detrended data are due to an increase of the actual values, we still take these results as further indicators that the predictability tends to be lower for more recent years.

### 4.3. Correlations Predictability/Complexity

We further searched for correlations between the predictability, i.e., the R2 cross-validation (R2CV) score and the R2 score on the test set (R2), and all calculated signal complexities. We thus found a relation between both ApEn and SampEn and both R2CV and R2. When it comes to differentiating between the M1-detrended and the non-detrended data, we found that they, for this relation, complement each other. We further fitted this relation using a generalized logistic function [60]:

(28) $$y\left(t\right)=A+\frac{K-A}{{\left(C+Qe^{-Bt}\right)}^{\frac{1}{\nu }}},$$

and curve_fit from the Python package scipy. Next, we performed a $\chi^{2}$ test to estimate the goodness of the fit, and check for significance, i.e., if the $\chi^{2}$ value is below 0.05.

**Figure 20:**
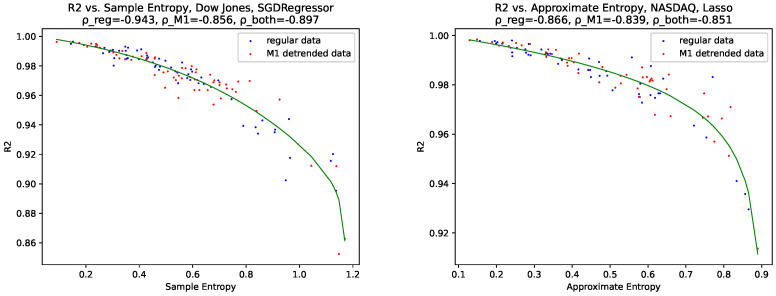
Best fits of the generalized logistic function for both data sets, the Dow Jones data and the NASDAQ data.

The relations/fits with the lowest $\chi^{2}$ values can be found in Figure 20. The plots for all relations, i.e., ApEn, SampEn, R2CV, R2, and all regressors can be found in Appendix A. All $\chi^{2}$ values can be found in Table 3 and Table 4.

When interpreting these findings, the M1-detrended data and the non-detrended data complement each other for the relationship between predictability and Ap- and SampEn can be explained such that predictability originates from the same process, i.e., always the same algorithm on a similar dataset. When it comes to the similarity of the data set in terms of the inherent complexity, we see that, taking into account our findings from Section 4.1 for ApEn and SampEn, that these entropies are very similar in terms of fluctuations when comparing the M1-detrended and the non-detrended data sets. Further, when thinking about the inherent information, we conclude that both data sets, the M1-detrended and the non-detrended, contain some aspects of the same process, i.e., stock data and the corresponding money supply. In addition, as discussed in Section 4.1, for Fisher’s information, for some years, the M1 adjustment increases the inherent information. For some years, it reduces the inherent information, but somehow keeps key features (high peaks, low peaks, and trends) intact, which can be seen in the Figure 9, i.e., Shannon’s entropy, and Figure 3, Figure 4 and Figure 5, i.e., all calculated fractal dimensions.

When it comes to the results of the $\chi^{2}$ fit, we see that all obtained $\chi^{2}$ values (Table 3 and Table 4) are very low. Thus the observed generalized logistic behavior is significant. Still, we give the corresponding critical values $\chi^{2}$ for a significance level of 0.05 and the corresponding degrees of freedom. As our generalized logistic function takes six parameters, we need to reduce the number of samples by 6+1 to obtain the degrees of freedom.

- **Dow Jones:** Degrees of freedom (The number of samples is always two times the number of years available for each data set, as we used both the M1-adjusted and the non-adjusted data) =122−7=115 and $\chi_{crit,\mathrm{Dow}\mathrm{Jones}}^{2}=141.03$;
- **NASDAQ:** Degrees of freedom =98−7=91 and $\chi_{crit,\mathrm{NASDAQ}}^{2}=114.27$.

Thus, we can conclude that both SampEn and ApEn are suitable measures for the predictability of stock market data for the tested machine learning algorithms. We, therefore, expected that machine learning algorithms perform with less accuracy on data sets with a high ApEn or SampEn value, i.e., above or close to 1 for both entropies than on datasets with lower ApEn or SampEn values, for the best cases around 0.1–0.2.

We further gave the correlation coefficients for each data set, i.e., the M1-detrended and the non-detrended, and both together, and as can be seen in plots Figure 20 and Appendix A. Thus we see that the correlation coefficients also indicate a strong correlation between ApEn, SampEn and R2CV and R2, as their values are consistently above 0.7.

### 4.4. Key Findings

Given the analysis of all employed methods above, we briefly summarize our key findings:

- We found a relation between ApEn/SampEn and the predictability of the employed ML algorithms. We found that we can model this relation using a generalized logistic function. Given the applied $\chi^{2}$ test results, we conclude that this relation holds for all algorithms and all data under study. Thus this relation states: High ApEn/SampEn indicates low predictability and vice versa.
- Shannon’s entropy shows an increase of disorder for both Dow Jones and NASDAQ and subsequently for both the M1-detrended and the non-detrended data. We conclude that the disorder in the data increased for later years. We can further see that the M1-adjustment increased Shannon’s entropy for earlier years for all data and thus conclude that it adds disorder to the data, and that later years are inherently more random, and that the disorder induced by M1-adjustment is already present in the data.
- The employed algorithms to calculate a fractal dimension and R/S analysis to calculate the Hurst exponent suggest that the stock market data under study became more random/complex for later years.
- Using the Hurst error, we found that later years for all years are closer to a fractional Brownian motion than earlier years, which is more apparent for the NASDAQ data. On the other hand, the Dow Jones data is closer to a fractional Brownian motion right from the start. Thus, we only observe a slight decrease towards fractional Brownian behavior.
- In general, the M1 adjustment led to decreasing predictability of the data under study, as can be seen in the tables for the average errors, i.e., Table 1 and Table 2. However, given the corresponding large errors and Figure 18 and Figure 19, we see that this is not true for all regions of the data, as there are some parts where the M1-detrending increased the predictability.
- Our analysis of the predictability of both data sets, i.e., Dow Jones and NASDAQ, and both the M1-detrended and the non-detrended data indicate lower predictability for later years.

## 5. Conclusions

We employed three machine learning algorithms and a range of complexity measures to detect trends in the predictability of stock market data, namely the Dow Jones Industrial Average and the NASDAQ Composite. We further adjusted the stock market data understudy for the M1 money supply to show its impact on predictability and complexity.

We found that the stock market data under study tends to be more random and unpredictable for later years than for earlier years. Further, given the results of the Hurst exponent, the corresponding error, and the employed fractal dimensions, we conclude that later data, e.g., 2010–2019, show more similarities with a fractional Brownian motion than earlier years. Here we consider our findings regarding low errors of the Hurst exponent fit, where very low errors suggest a behavior close to a fractional Brownian motion. Moreover, an increase towards a Hurst exponent of ≈0.5, or fractal dimensions of ≈1.5, suggests a random behavior. We observe these trends for both the Dow Jones and the NASDAQ data, however the trends are more prominent for the NASDAQ data.

We further observed that the M1 detrending increases the randomness in data sets for earlier years when analyzed using Shannon’s entropy. For later years, the M1 adjustment does not add additional noise to the data, which we interpret such that the varying money supply already influences data.

Further, the M1 adjustment slightly decreases the predictability for both data sets under study. This is another indication that the M1-detrending adds noise to the data instead of removing it. Here the authors assume that this effect may be diminished for the most recent years and suggest that this should be the topic of future research.

As this study aims to show the predictability of stock market data, however not to test which algorithm performs best, our results for comparing the three employed algorithms should not be taken as evidence that Lasso regression outperforms the other employed algorithms. We did not predict future trends, etc., but performed a regression analysis on the data sets under study.

Moreover, we found evidence that approximate entropy and sample entropy, two entropy measures specifically designed for time-series data, indicate the performance of a data set in machine learning regression analysis. Here, for both entropy measures, we observed that a high entropy indicates low predictability and vice versa.

We suggest more research to be done on the link between machine learning regression analysis, approximate entropy, and sample entropy, as this correlation may be exploited to find stock market periods with high predictability. In addition, one may find noisy regimes in any data set. Thus this finding may improve regression analysis approaches on any data set. Further, given the literature mentioned at the beginning and our findings, we aim to motivate researchers to employ complexity measures and ideas from Chaos theory to improve their machine learning approaches.

The last point to discuss is if machine learning and deep learning approaches can effectively predict stock market data. Our presented results show that with increasing complexity in terms of ApEn and SampEn, the performance of the employed algorithms is reduced. In [16], the researchers suggest that periods of lower complexity in terms of the Hurst exponent, i.e., periods that are more persistent, can be predicted with higher accuracy using neural networks. Similar results are presented in [11] i.e., periods of the Dow Jones Industrial Average with increased Hurst exponents can be predicted with higher accuracy than periods with Hurst exponent’s closer to H=0.5 using neural networks. In [15], R/S analysis, i.e., the Hurst exponent and the fractal dimension of a time series data are employed as a fractal analysis for predicting exchange rates. Fractal analysis shows that the considered exchange rates can be forecast. Next, the considered data is forecast using tree-based algorithms. Summing up these findings and the presented research in this article, the authors conclude that measures of signal complexity can be employed to identify predictable periods in stock market data, i.e., periods of comparatively low complexity or high persistency. These periods are forecastable with higher accuracy than periods with opposing characteristics, i.e., increased complexity and anti-persistency.

## Figures and Tables

**Table 1:** Average errors for each algorithm for the Dow Jones data set.

	R2CV Reg	R2CV M1	R2 Reg	R2 M1	RMSE reg	RMSE M1
XGBoost	0.9604 ± 0.031	0.9575 ± 0.0342	0.9727 ± 0.0213	0.9696 ± 0.0277	41.2794 ± 47.5003	3.4495 ± 2.0942
Lasso	0.9641 ± 0.0306	0.9624 ± 0.0342	0.9751 ± 0.0221	0.975 ± 0.0244	41.6535 ± 51.1356	3.2242 ± 1.8485
SGD	0.9609 ± 0.033	0.9583 ± 0.0356	0.9711 ± 0.0244	0.9706 ± 0.0265	45.5918 ± 54.1015	3.7737 ± 2.1923

**Table 2:** Average errors for each algorithm for the NASDAQ data set.

	R2CV Reg	R2CV M1	R2 Reg	R2 M1	RMSE reg	RMSE M1
XGBoost	0.9734 ± 0.0211	0.9678 ± 0.0263	0.9811 ± 0.0185	0.9779 ± 0.0214	15.476 ± 18.4906	1.4357 ± 1.8687
Lasso	0.9777 ± 0.0209	0.9736 ± 0.0228	0.9837 ± 0.0152	0.9827 ± 0.0148	14.7437 ± 18.2027	1.3279 ± 1.7913
SGD	0.9754 ± 0.0209	0.9702 ± 0.0248	0.9794 ± 0.0195	0.9782 ± 0.017	16.996 ± 20.2584	1.564 ± 1.9638

**Table 3:** Table for the $\chi^{2}$ values for each logistic fit for the Dow Jones data set.

	$\chi^{2}$			$\chi^{2}$
R2CV—ApEn—XGBoost	0.0134		R2—ApEn—XGBoost	0.0147
R2CV—SampEn—XGBoost	0.0129		R2—SampEn—XGBoost	0.0145
R2CV—ApEn—Lasso	0.0115		R2—ApEn—Lasso	0.0100
R2CV—SampEn—Lasso	0.0109		R2—SampEn—Lasso	0.0094
R2CV—ApEn—SGDRegressor	0.0103		R2—ApEn—SGDRegressor	0.0080
R2CV—SampEn—SGDRegressor	0.0090		**R2—SampEn—SGDRegressor**	** 0.0077 **

**Table 4:** Table for the $\chi^{2}$ values for each logistic fit for the NASDAQ data set.

	$\chi^{2}$			$\chi^{2}$
R2CV—ApEn—XGBoost	0.0050		R2—ApEn—XGBoost	0.0041
R2CV—SampEn—XGBoost	0.0068		R2—SampEn—XGBoost	0.0059
R2CV—ApEn—Lasso	0.0029		**R2—ApEn—Lasso**	** 0.0022 **
R2CV—SampEn—Lasso	0.0050		R2—SampEn—Lasso	0.0030
R2CV—ApEn—SGDRegressor	0.0023		R2—ApEn—SGDRegressor	0.0030
R2CV—SampEn—SGDRegressor	0.0043		R2—SampEn—SGDRegressor	0.0048

**Figure A1:**
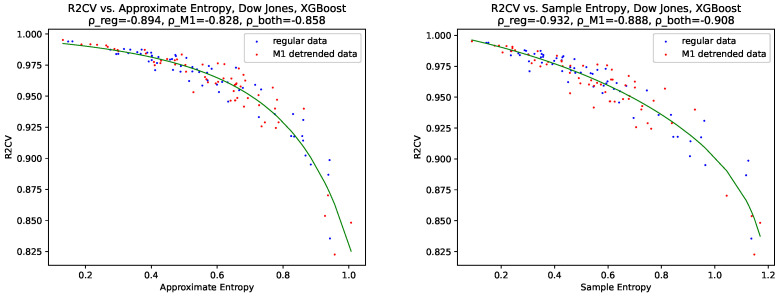
Dow Jones predictability of R2 cross validation vs. ApEn and SampEn for XGBoost.

**Figure A2:**
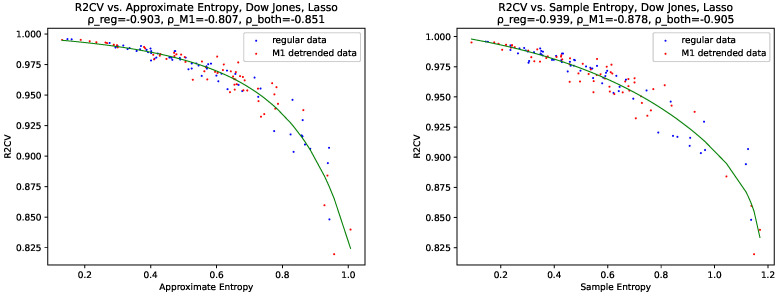
Dow Jones predictability of R2 cross validation vs. ApEn and SampEn for Lasso regression.

**Figure A3:**
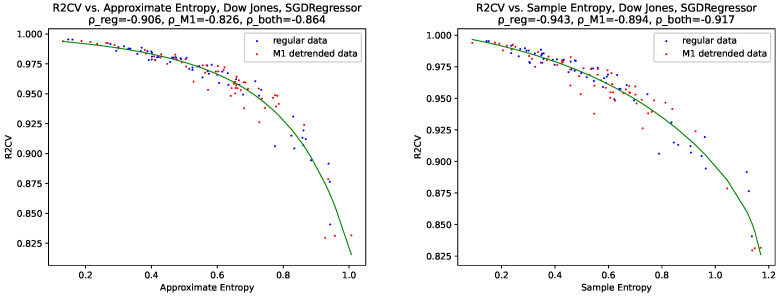
Dow Jones predictability of R2 cross validation vs. ApEn and SampEn for the SGD regressor.

**Figure A4:**
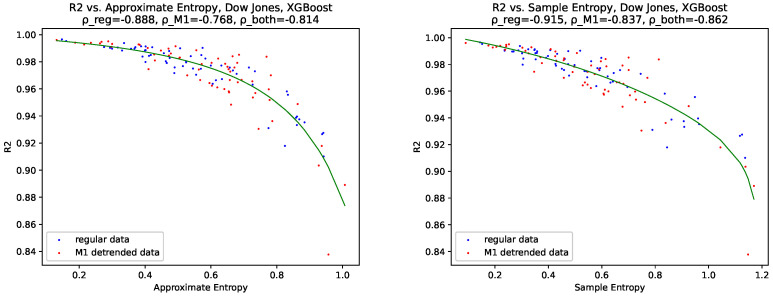
Dow Jones predictability of R2 score on the test data vs. ApEn and SampEn for XGBoost.

**Figure A5:**
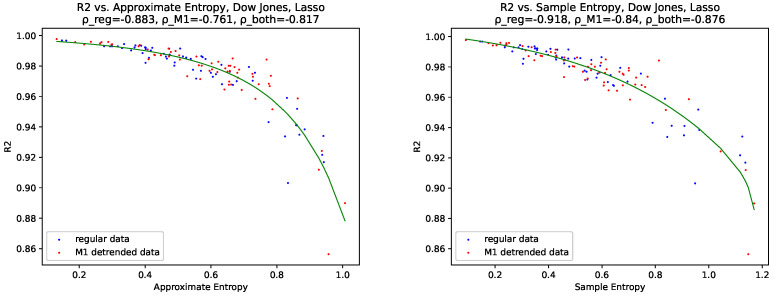
Dow Jones predictability of R2 score on the test data vs. ApEn and SampEn for Lasso regression.

**Figure A6:**
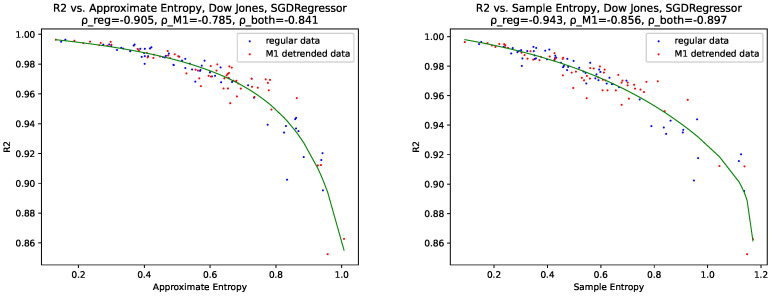
Dow Jones predictability of R2 score on the test data vs. ApEn and SampEn for the SGD regressor.

**Figure A7:**
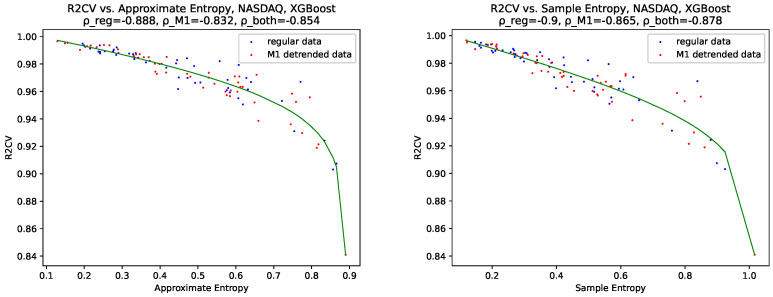
NASDAQ predictability of R2 cross validation vs. ApEn and SampEn for XGBoost.

**Figure A8:**
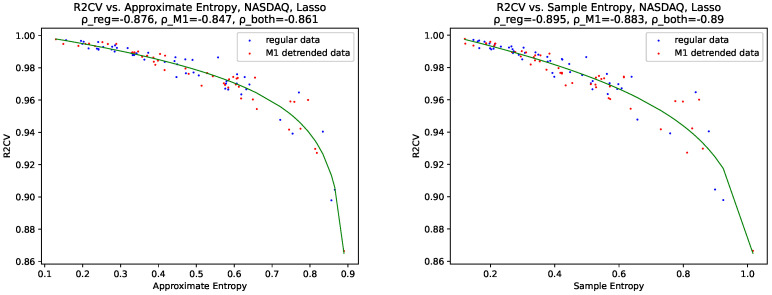
NASDAQ predictability of R2 cross validation vs. ApEn and SampEn for Lasso regression.

**Figure A9:**
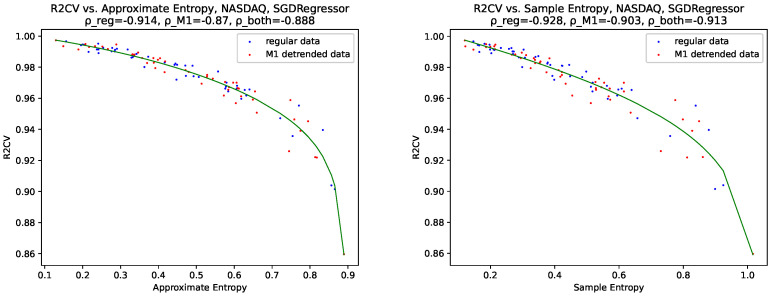
NASDAQ predictability of R2 cross validation vs. ApEn and SampEn for the SGD regressor.

**Figure A10:**
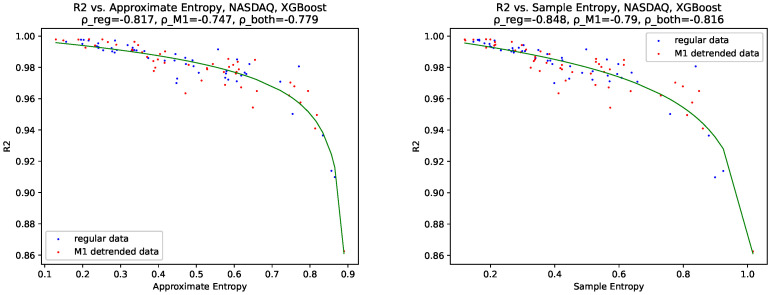
NASDAQ predictability of R2 score on the test data vs. ApEn and SampEn for XGBoost.

**Figure A11:**
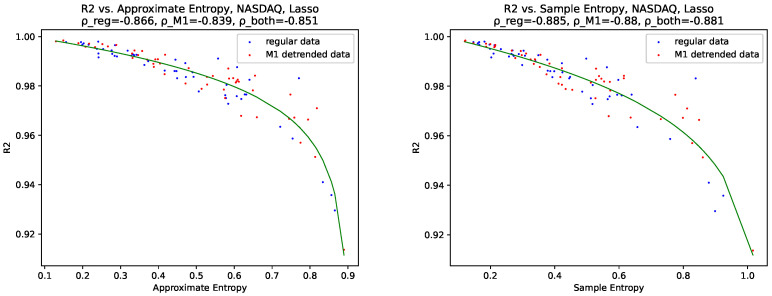
NASDAQ predictability of R2 score on the test data vs. ApEn and SampEn for Lasso regression.

**Figure A12:**
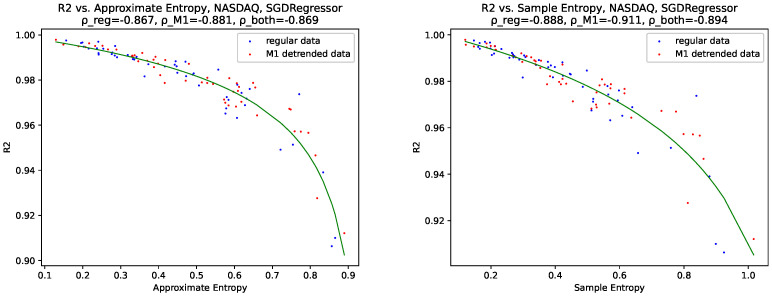
NASDAQ predictability of R2 score on the test data vs. ApEn and SampEn for the SGD regressor.

**Figure A13:**
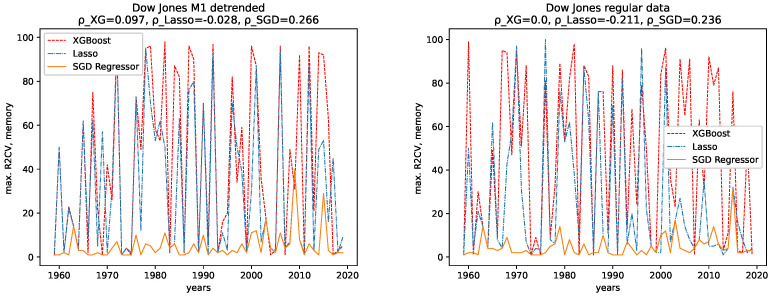
Plots for the memory, i.e., input of each algorithm for maximal R2CV score, Dow Jones data set.

**Figure A14:**
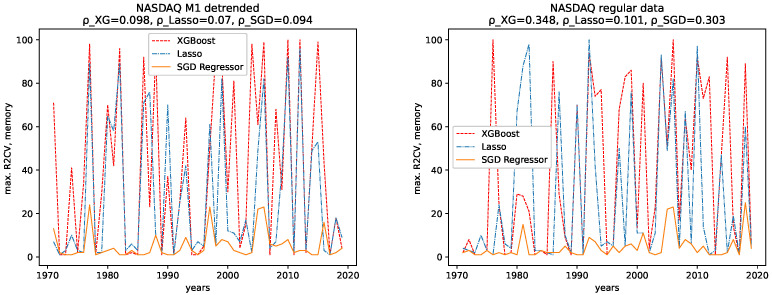
Plots for the memory, i.e., input of each algorithm for maximal R2CV score, NASDAQ data set.

**Figure A15:**
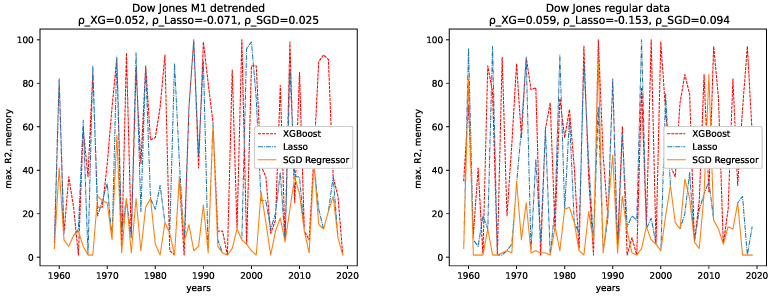
Plots for the memory, i.e.,m input of each algorithm for maximal R2 score, Dow Jones data set.

**Figure A16:**
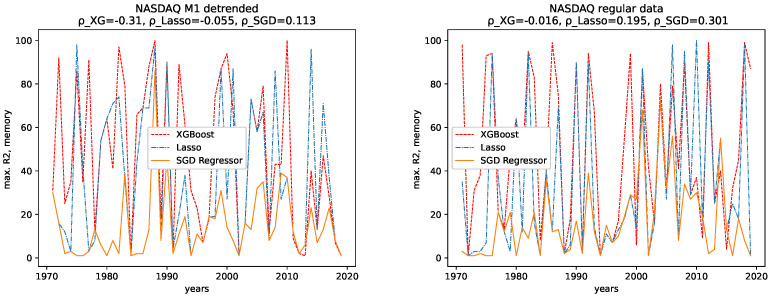
Plots for the memory, i.e., input of each algorithm for maximal R2 score, NASDAQ data set.

**Figure A17:**
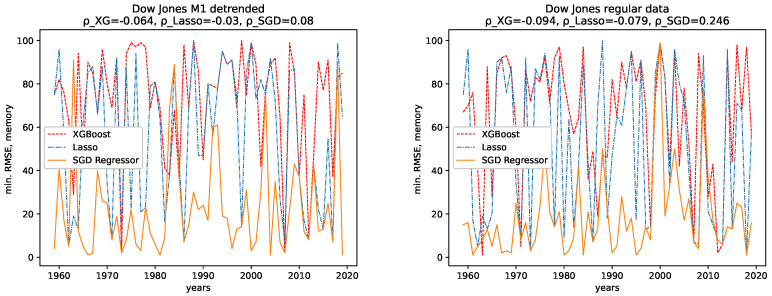
Plots for the memory, i.e., input of each algorithm for minimal RMSE, Dow Jones data set.

**Figure A18:**
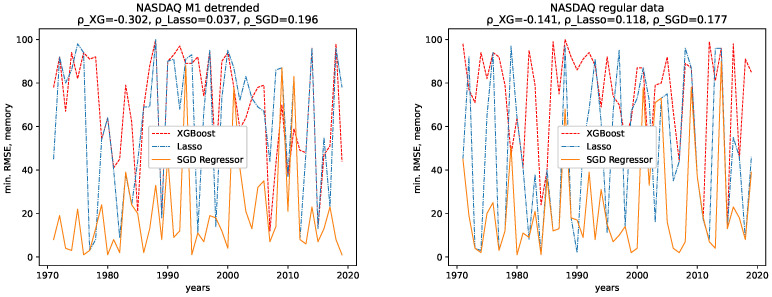
Plots for the memory, i.e., input of each algorithm for minimal RMSE, NASDAQ data set.

**Figure A19:**
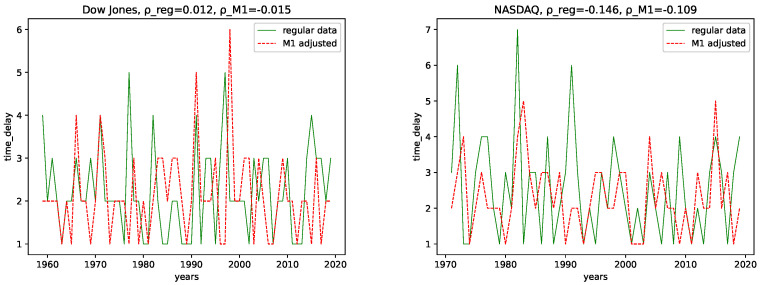
Plots for the time delay for each year.

**Figure A20:**
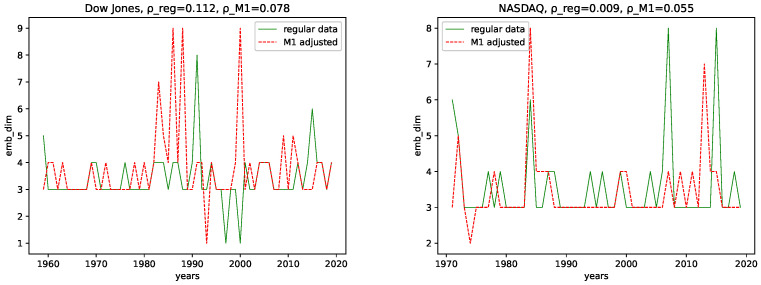
Plots for the embedding dimension for each year.

## Data Availability

Not applicable.

## Appendix A. ApEn and SampEn vs. Predictability

Here we show the additional correlation between ApEn SampEn and the R2-scores.

## Appendix B. Memory Plots

Here we show the memory, i.e., the consecutive time steps taken as each algorithm’s input. We can hardly see any trends here, as the calculated correlation coefficients show. The only significant finding here is that the SGD regressor, which has overall has the worst performance, taking a lower number of input data steps for maximal performance. Thus, we conclude that there is a memory in stock market data, however, that is still a vague claim, and further research needs to be done on the subject.

## Appendix C. Time Delay and Embedding Dimensions

For each data set, we calculated the time delay using the method for mutual information.
